# Clinical Analysis of *Serratia* Species Infections in Children and Adolescents Treated for Cancer or Undergoing Hematopoietic Stem Cell Transplantation—A Multicenter Nationwide Study

**DOI:** 10.3390/pathogens15070725

**Published:** 2026-07-09

**Authors:** Ewelina Truszkowska, Małgorzata Salamonowicz-Bodzioch, Jowita Frączkiewicz, Krzysztof Kałwak, Filip Pierlejewski, Małgorzata Nowak, Maciej Zdunek, Wojciech Młynarski, Krzysztof Czyżewski, Kamila Jaremek, Oliwia Grochowska, Patrycja Zalas-Więcek, Katarzyna Derwich, Weronika Solpa, Karolina Baranowska, Agnieszka Mizia-Malarz, Olga Gryniewicz-Kwiatkowska, Magdalena Łukszo, Bożenna Dembowska-Bagińska, Ewa Bień, Ninela Irga-Jaworska, Jan Styczyński, Olga Zając-Spychała

**Affiliations:** 1 Department of Pediatric Oncology, Hematology and Transplantology, Institute of Pediatrics, Poznan University of Medical Sciences, 60-572 Poznan, Poland; 2 Department of Pediatric Stem Cell Transplantation, Hematology and Oncology, Wroclaw Medical University, 50-556 Wroclaw, Poland; 3 Department of Pediatrics, Oncology and Hematology, Medical University of Lodz, 91-738 Lodz, Poland; 4 Department of Pediatrics, Haematology, Oncology, Immunology and Transplantology, Collegium Medicum in Bydgoszcz, Nicolaus Copernicus University, 85-094 Bydgoszcz, Poland; 5 Department of Microbiology, Collegium Medicum in Bydgoszcz, Nicolaus Copernicus University, 85-094 Bydgoszcz, Poland; 6 Department of Oncology, Hematology and Chemotherapy, Upper Silesian Child’s Health Care, Medical University of Silesia, 40-752 Katowice, Poland; 7 Department of Oncology, The Children’s Memorial Health Institute, 04-730 Warsaw, Poland; 8 Department of Pediatrics, Hematology and Oncology, Medical University of Gdansk, 80-952 Gdansk, Poland

**Keywords:** pediatric oncology, *Serratia*, antibiotic resistance

## Abstract

*Serratia* species are Gram-negative pathogens responsible for a wide range of nosocomial infections. This multicenter nationwide retrospective study aimed to describe the epidemiology, clinical characteristics, antimicrobial susceptibility, and outcomes of *Serratia* infections in pediatric oncology patients and hematopoietic stem cell transplantation (HSCT) recipients in Poland between 2012 and 2023. A total of 36 *Serratia* infection episodes were identified in patients under 20 years of age, including 30 cases (83.3%) in the oncological (OHD) group and six (16.7%) among HSCT recipients. The median age was 4.30 years. The most common underlying diseases were acute lymphoblastic leukemia (36.1%) and central nervous system tumors (16.7%). Bloodstream infections predominated in OHD patients (33.3%), whereas urinary tract infections were most frequent in HSCT recipients (83.3%). *S. marcescens* was the most commonly isolated species. More than half of isolates (53.3%) showed antimicrobial resistance, with extended-spectrum β-lactamase (ESBL)-producing strains in 26.7% and AmpC β-lactamase-producing strains in 13.3%. Multidrug resistance occurred in 30%. Treatment most often included amikacin, piperacillin/tazobactam, and carbapenems. Five deaths occurred in the OHD group and one in the HSCT group, none directly related to *Serratia* infection. Although uncommon, *Serratia* infections remain clinically relevant due to their high antimicrobial resistance, underscoring the need for antimicrobial stewardship.

## 1. Introduction

*Serratia* is a genus of Gram-negative bacteria which belong to the *Enterobacterales* order, the *Yersiniaceae* family. Certain *Serratia* species are well known for producing a red pigment called prodigiosin, which played a key role in the discovery of the genus in the 19th century [1]. In Italy, peasants who lived near Padua observed that polenta (dish made with cornmeal) turned red. Initially, they believed that it was caused by supernatural powers. Later, a commission which included professors from a nearby University of Padua investigated the topic; however, it was an independent researcher, the pharmacist Bartolomeo Bizio, who ultimately found the cause. He named the newly discovered organism after the Italian scientist, Serafino Serati, hence *Serratia*, although he thought that it was a fungus [2]. Now, we know that only three *Serratia* species produce prodigiosin: the most common *S. marcescens*, *S. plymuthica* and *S. rubidaea* [3]. Besides providing color, prodigiosin also exhibits a range of biological properties, increasing its virulence, for instance, acting against competing microbes [1]. To date, 23 species belong to the *Serratia* genus, including six species of clinical significance in humans: *S. marcescens*, *S. liquefaciens*, *S. plymuthica*, *S. rubidaea*, *S. odorifera*, and *S. fonticola* [4]. However, for many years, *Serratia* was considered non-pathogenic, despite sporadic reports of infections, and was even used as a tracer organism in medical and military experiments. Currently, we are aware that *Serratia* causes a wide range of infections, including sepsis, bacteriemia, urinary tract infections, meningitis, respiratory tract infections and wound infections. Eye infections of *S. marcescens* origin are also common, especially among patients who use contact lenses. Together with *Pseudomonas aeruginosa* it is the most often reported Gram-negative bacterium when ocular infections in contact lenses users are concerned [2]. *S. marcescens* is well known for causing outbreaks in hospital settings, especially in neonatal intensive care units (NICU) [5]. The first outbreak among the pediatric population was reported by Rabinowitz and Schiffrin in 1952 and involved 12 pediatric patients. One of them, a neonate, died due to meningitis. Notably, this outbreak was the first to be traced to a point source—a contaminated intravenous solution [2]. The literature distinguishes various sources of *S. marcescens*, including obvious ones like the hands of health care workers, contaminated breast milk, formula, medical equipment as well as unusual ones like contaminated hand brushes or cotton wool pads. One outbreak in a pediatric oncology unit, as described in the literature, was reported by McAllister et al. in 1986 [6]. Twelve episodes of *S. marcescens* bacteremia occurred in 10 patients over an 18-month period, resulting in three deaths. The outbreak was linked to contaminated aqueous chlorhexidine stored in a bedside container containing plastic clamps. Where antibiotic resistance is concerned, *S. marcescens* is naturally resistant to many antibiotics, including ampicillin, first-generation cephalosporins, macrolides and colistin. Isolates may also develop several acquired resistance mechanisms such as extended-spectrum β-lactamase (ESBL), carbapenemases, aminoglycoside-modifying enzymes and fluoroquinolone resistance [1].

The aim of this multicenter nationwide study was to describe the epidemiology, clinical characteristic, antimicrobial susceptibility pattern and outcome of *Serratia* infections in pediatric cancer patients and hematopoietic stem cell transplantation (HSCT) recipients in Polish pediatric hematology and oncology centers and pediatric HSCT centers, over a period of 12 years (2012–2023).

## 2. Materials and Methods

Design of the study: This was a retrospective multicenter study. Due to its retrospective nature, the requirement for obtaining informed consent from individual patients was waived. Medical records were collected from all Polish pediatric oncology and transplant centers and analyzed centrally. All patients were treated according to contemporary chemotherapy protocols, and all HSCTs were performed in accordance with institutional procedures and treatment guidelines.

Patients: Between 2012 and 2023, a total of 11,404 children with cancer were included in the analysis, and 2037 HSCT procedures were performed. In the oncohematological diseases (OHD) group, 15,662 infectious episodes were reported, including 10,893 bacterial infections. Among HSCT recipients, 4180 infectious episodes were recorded, of which 1826 were bacterial.

Antimicrobial prophylaxis: Standard antimicrobial prophylaxis was administered to all patients, including trimethoprim/sulfamethoxazole (TMP/SMX) given three times weekly for prophylaxis against *Pneumocystis jirovecii*. Standard non-pharmacological infection-prevention measures were also implemented, including hand hygiene before patient contact and the use of maximal sterile barrier precautions during central venous catheter insertion. Patients with acute myeloid leukemia (AML) who developed neutropenia received center-specific antibacterial prophylaxis. A similar approach was applied to HSCT recipients, who also received center-specific antibacterial prophylaxis. Because prophylactic regimens were administered according to standards in effect at the time of treatment, they varied depending on both the underlying diagnosis and the treatment period. For antifungal prophylaxis, fluconazole was used until 2016, after which posaconazole was introduced for patients with leukemia and HSCT recipients. Antiviral prophylaxis with acyclovir was routinely administered to HSCT recipients.

Microbiological testing: Microbiological cultures were done in local accredited microbiology laboratories at all participating centers. Specimens were processed according to standard local protocols in place at the time of testing.

*Serratia* spp. isolates were identified to the species level using routine identification methods available at the participating laboratories, predominantly mass spectrometry in the MALDI Biotyper system (Bruker Daltonics GmbH, Bremen, Germany). Antimicrobial susceptibility testing was performed using routine automated susceptibility testing systems employed at the participating laboratories, including NMIC panels read with the Phoenix M50 system (Becton Dickinson, Franklin Lakes, NJ, USA), and interpreted according to the European Committee on Antimicrobial Susceptibility Testing (EUCAST) clinical breakpoints applicable in the respective year. *Serratia* spp. isolates were classified as ESBL-producers based on their resistance to penicillins and extended-spectrum cephalosporins, and DDST (double-disk synergy test). Where applied, cefepime disks were added to increase the sensitivity of ESBL detection. Detection was performed using standard techniques, for instance Mueller–Hinton agar supplemented with cloxacillin (bioMérieux, Marcy-l’Étoile, France). In the absence of susceptibility of the strains to at least one of the carbapenems (i.e., imipenem, meropenem or ertapenem), various tests were performed, including Carba NP test (Bufor B-PER II—Thermo Scientific, Waltham, MA, USA; Tienam (imipenem 500 mg + cilastatin 500 mg)—Merck Sharp & Dohme, Rahway, NJ, USA; 0.5% Phenol-red solution—Sigma Aldrich, St. Louis, MO, USA; and ZnSO4_7H2O—Merck Sharp & Dohme, Rahway, NJ, USA). To detect the type of carbapenemase, phenotypic tests were applied.

MDR bacteria were defined as isolates non-susceptible to one or more agents in three or more antimicrobial classes, XDR bacteria as isolates non-susceptible to one or more agents in all but two or fewer classes, and PDR (pan drug resistant) bacteria as non-susceptible to all antimicrobial classes tested.

Statistical Analysis: Descriptive statistics were presented as absolute numbers (*n*) and percentages (%) for categorical variables. Continuous variables were summarized as median values with ranges. Due to the small number of isolates, further statistical analyses were not performed. Comparisons between groups were limited to Fisher’s exact test.

## 3. Results

### 3.1. Demographics and Incidence

Over a period from 2012 to 2023 a total of 36 episodes of *Serratia* species infections were reported in children and adolescents < 20 years treated in Polish pediatric hematology and oncology centers who were enrolled into the retrospective, multicenter nationwide study. Infections occurred in 14 girls and 22 boys with median age of 4.30 years (range: 0.74–19.93 years). The most common diagnoses of infected patients were: acute lymphoblastic leukemia (ALL) (*n* = 13; 36.1%) and CNS (central nervous system) tumors (*n* = 6; 16.7%). Infections were subdivided into oncohematological diseases (OHD) group (*n* = 30; 83.3%) and HSCT group (*n* = 6; 16.7%). The characteristics of the analyzed group of patients is given in Table 1. During the study period, within the analyzed oncohematological diseases (OHD) subgroup (without HSCT), a total of 30 *Serratia* infections occurred in 13 girls and 17 boys with median age of 4.65 years (range: 1.17–19.93 years). The most common diagnoses of infected patients were: ALL (*n* = 10; 33.3%) and CNS tumors (*n* = 6; 20%) (Table 2). During the study period, within the analyzed HSCT subgroup a total of six *Serratia* spp. infections occurred in one girl and five boys with median age of 3.32 years (range: 0.74–10.24). The most common underlying diseases that were indications for HSCT among infected patients were ALL and AML (both *n* = 2; 33.3%). The characteristics of analyzed groups are given in Table 1 and Table 2.

### 3.2. Clinical and Microbiological Characteristics

Among the 30 infection episodes in OHD patients, bloodstream infections were the most common presentation (*n* = 10, 33.3%), followed by isolates detected in stool samples (*n* = 8, 26.7%) and urine (*n* = 5, 16.7%). The most frequently identified species were *S. marcescens* (*n* = 20, 66.7%) and *S. liquefaciens* (*n* = 7, 23.3%). Among the six infection episodes in HSCT recipients, isolates were most commonly recovered from urine (*n* = 5, 83.3%), followed by stool (*n* = 1, 16.7%). The predominant species was *S. marcescens* (*n* = 5, 83.3%), while *S. ficaria* was identified in one case (16.7%). Microbiological and clinical characteristics of *Serratia* infections in both groups are summarized in Table 3.

### 3.3. Antimicrobial Susceptibility and Resistance

In the OHD subgroup, 53.3% of isolates were resistant to at least one tested antibiotic. The most frequently observed mechanism was ESBL production (*n* = 8, 26.7%), including *S. liquefaciens* (*n* = 6, 20.0%) and *S. marcescens* (*n* = 2, 6.7%). Resistance to TMP/SMX was the second most common finding (*n* = 7, 23.3%), followed by resistance to gentamicin and ciprofloxacin (both *n* = 6, 20.0%). AmpC β-lactamase production was identified in four isolates (13.3%). Multidrug resistance was observed in nine isolates (30.0%). These isolates exhibited combined resistance mechanisms, including ESBL or AmpC production together with resistance to additional antibiotics. The most common multidrug-resistance pattern was ESBL or AmpC production combined with resistance to gentamicin, ciprofloxacin, and TMP/SMX (*n* = 4, 13.3%). In the HSCT subgroup, antimicrobial resistance was identified in two isolates (33.3%). One isolate was resistant to TMP/SMX and cephalosporins, while another AmpC-producing isolate showed resistance to first-, second-, and third-generation cephalosporins as well as piperacillin/tazobactam. Susceptibility data were not available for two patients. To assess temporal trends in antimicrobial resistance, the study period was divided into two intervals (2012–2017 and 2018–2023). Both OHD patients and HSCT recipients were included in this analysis. The proportion of resistant isolates was 60.0% (12/20) in the first period and 42.9% (6/14) in the second period. No statistically significant difference was observed between the two periods (Fisher’s exact test, *p* = 0.487).

### 3.4. Antibiotic Therapy Applied

A variety of antibiotic regimens was used in OHD patients, including monotherapy and combination therapy. Monotherapy was used in 13 episodes (43.3%), two-drug combinations in nine (30.0%), three-drug combinations in four (13.3%), and four-drug combinations in three (10.0%). One patient, who had an AmpC-producing *S. marcescens* isolate identified in the bronchoalveolar lavage, did not receive targeted antimicrobial therapy. The most frequently used antibiotics were amikacin (*n* = 13, 43.3%), piperacillin/tazobactam (*n* = 12, 40.0%), and meropenem (*n* = 9, 30.0%). Antibiotic selection was primarily empirical and based on the patient’s clinical condition, laboratory findings, and the treating physician’s judgment at the time of infection. In most cases, therapy was initiated empirically and subsequently adjusted according to clinical response and microbiological results when available. Treatment was modified in four cases (13.3%) due to clinical or microbiological findings, including febrile neutropenia, typhlitis, or antibiogram results. Among HSCT recipients, antibiotic therapy also consisted of a variety of regimens, including monotherapy in three cases (50.0%) and two-drug combinations in two (33.3%). Data were unavailable for one patient with *S. ficaria* infection. Carbapenems (meropenem and imipenem) were used in three cases (50.0%), and cefepime in two (33.3%). Teicoplanin was added once to meropenem, and neomycin was added once to cefepime. Due to the retrospective and multicenter design of the study, detailed information regarding exact dosing regimens and duration of therapy was not uniformly available and therefore could not be analyzed.

### 3.5. Treatment Outcomes

In OHD patients, five deaths were recorded; none were directly attributed to *Serratia* spp. infection. The leading cause of death was disease progression (*n* = 4, 80.0%), followed by typhlitis and its complications (*n* = 1, 20.0%). Infections contributed to mortality in two cases. Acute myeloid leukemia or MDS/AML was the most common underlying diagnosis among patients who died (*n* = 3, 60.0%). Among HSCT recipients, one patient with neuroblastoma died due to sepsis and veno-occlusive disease. Further details regarding causes of death in individual patients are presented in Table 4.

## 4. Discussion

We report 36 *Serratia* infections from all Polish pediatric hematology and oncology centers over the period 2012–2023, which is consistent with the limited data available in the literature, including previous Polish analyses. A single-center study from Kraków by Klepacka et al. [7] reported only two cases of *Serratia marcescens*, despite analyzing 7667 blood samples collected from 1914 patients between 2011 and 2021. Additionally, Zawitkowska et al. [8] reported one case of *S. marcescens* in a national multicenter study; however, that study focused exclusively on patients with ALL treated between 2020 and 2021. Single-center studies from pediatric oncohematology departments in other countries show similar findings. For example, in a Turkish single-center study by Kara et al. [9], five cases of *S. marcescens* were identified between 2010 and 2015. In contrast, an Italian multicenter study including 22 centers (2018–2019) [10] did not report any *Serratia* infections among bloodstream infections. A similar study from Verona by Mattei et al. [11] also did not distinguish *Serratia* species; it included patients who required hospitalization for intravenous broad-spectrum antibiotic therapy over a longer period (2010–2019).

A notable finding of our study was the high prevalence of antimicrobial resistance, observed in more than half of the analyzed cases (52.9%). Our findings are consistent with those of the previously mentioned study by Kara et al. [9], which also demonstrated a high level of antimicrobial resistance, with only 60% of isolates susceptible to meropenem and no susceptibility to piperacillin/tazobactam or colistin. No significant change in antimicrobial resistance was observed over time. Although resistant isolates were less frequent in the later study period, the difference was not statistically significant, likely due to the small sample size.

We identified a substantial proportion of ESBL-producing (26.7%) and AmpC-producing (13.3%) *Serratia* strains. ESBLs are enzymes that inactivate most penicillins, cephalosporins, and aztreonam. The 2024 Infectious Diseases Society of America (IDSA) guidelines [12] for the management of antimicrobial-resistant Gram-negative infections recommend carbapenems as first-line therapy for ESBL-producing infections outside the urinary tract. Despite their frequent use in oncological patients, piperacillin/tazobactam is not recommended even when in vitro susceptibility is reported. First, susceptibility testing and MIC values may be unreliable in the presence of ESBLs and other β-lactamases, such as OXA-1. Second, the risk of bacterial regrowth appears higher with piperacillin/tazobactam than with meropenem. However, in selected clinical scenarios, TMP/SMX and fluoroquinolones may be considered, particularly for complicated urinary tract infections and pyelonephritis, or as step-down therapy within a carbapenem-sparing strategy.

AmpC β-lactamases are commonly produced by Gram-negative *Enterobacterales* and represent one of the earliest mechanisms of penicillin resistance [13]. They may be constitutively or inducibly expressed and can be encoded chromosomally or on plasmids. Derepression of the ampC gene may lead to an increased production of AmpC β-lactamases during antibiotic therapy, resulting in the emergence of resistance despite initial susceptibility. The frequency of this mechanism varies among *Enterobacterales* and is estimated to be <5% in *Serratia marcescens*. AmpC β-lactamases hydrolyze cephalosporins and monobactams; however, cefepime is an exception due to its zwitterionic structure, which enables rapid penetration of the bacterial outer membrane and reduced susceptibility to enzymatic hydrolysis [14]. Carbapenems remain stable against AmpC-mediated hydrolysis, although clinical data are limited. As with ESBL-producing pathogens, piperacillin/tazobactam is not recommended for AmpC-producing *Enterobacterales* due to inferior clinical outcomes. Piperacillin is a β-lactam antibiotic, while tazobactam provides insufficient protection against AmpC-mediated hydrolysis. Treatment remains a challenge. Carbapenems are a reasonable option; however, their overuse may contribute to further antimicrobial resistance and the loss of last-resort agents. IDSA guidelines suggest cefepime as an alternative, while TMP/SMX and fluoroquinolones are recommended for urinary tract infections. In contrast, European Society of Clinical Microbiology and Infectious Diseases (ESCMID) guidelines [15] do not provide separate recommendations for ESBL- and AmpC-producing organisms and highlight the limited quality of available evidence.

This variability is reflected in our data, as patients received different antibiotic regimens. Notably, piperacillin/tazobactam was the second most frequently used antibiotic in the OHD group despite the above recommendations. However, in some cases, antimicrobial therapy was adjusted based on susceptibility testing results. Regarding mortality, no deaths were directly attributed to *Serratia* infection. In the literature, an outbreak described by McAllister et al. [6] resulted in three deaths; however, these occurred in the 1980s, when pediatric oncology and supportive care were less advanced than today. The main limitation of our study is its retrospective design. Due to the multicenter nature of the study and the long study period covering over 10 years (2012–2023), not all diagnostic, microbiological, and therapeutic procedures could be fully standardized across participating centers, and clinical management and laboratory practices changed over time in accordance with evolving local protocols and international guidelines. Moreover, because the analysis focused on infection episodes rather than individual patients, the clinical impact of recurrent infections could not be assessed. Also, all cultures positive for *Serratia* spp. were included in the analysis. No distinction was made between colonization and active infection. Although *Serratia* spp. isolates recovered from bronchoalveolar lavage fluid, endotracheal aspirates, and catheter tips at bacterial loads below 10^4^ CFU/mL, 10^6^ CFU/mL, and 10^3^ CFU/mL, respectively, may be considered indicative of colonization, as are isolates recovered from stool samples, quantitative microbiological data (e.g., bacterial loads expressed as CFU/mL) were not available for all collected specimens. Given the immunocompromised nature of the study population, *Serratia* colonization may also represent a clinically relevant finding. Moreover, the relatively small number of cases limited the statistical analysis to mainly descriptive methods and precluded multivariable analyses. Due to the small number of isolates, no statistical tests other than Fisher’s exact test were performed. Therefore, the results should be interpreted with caution. Further multicenter studies, including cohorts from other countries, are needed. In addition, antimicrobial prophylaxis could not be fully standardized due to the retrospective multicenter design and changes in treatment protocols over the study period. Pharmacological prophylaxis evolved over time according to contemporary standards and varied depending on both the underlying diagnosis and treatment period. Despite these limitations, the study has several strengths. It is a nationwide, multicenter analysis conducted over a 12-year period, including patients managed according to standardized, internationally endorsed treatment protocols. To our knowledge, this is the first comprehensive national analysis of *Serratia* infections in pediatric oncology patients and HSCT recipients in Poland.

## 5. Conclusions

Our findings show that, despite their rarity, *Serratia* infections represent a clinically relevant problem in an already highly burdened patient population due to high rates of multidrug resistance. Close collaboration between microbiologists and oncologists is essential to ensure appropriate antimicrobial therapy. As with other multidrug-resistant Gram-negative pathogens such as *Pseudomonas* and *Klebsiella*, management of *Serratia* infections requires a strong antimicrobial stewardship approach.

## Figures and Tables

**Table 1:** Baseline characteristics of oncohematological diseases (OHD) group and hematopoietic stem cell transplant (HSCT) group.

Characteristics	Total	OHD Group	HSCT Group
Number of episodes	36	30	6
Sex			
Female	14	13	1
Male	22	17	5
Median age at infection	4.30	4.65	3.32
Deaths in general	6	5	1

**Table 2:** Characteristics of underlying disease in oncohematological diseases (OHD) group and hematopoietic stem cell transplantation (HSCT) group.

Diagnosis	Number of Episodes (OHD Group; *n* = 30)	Number of Episodes (HSCT Group; *n* = 6)
Hematological disorders		
Acute lymphoblastic leukemia	10	2
Acute myeloid leukemia	5	2
MDS/AML	1	0
Juvenile myelomonocytic leukemia	0	1
Solid tumors		
Central nervous system tumors	6	0
Neuroblastoma	3	1
Hepatoblastoma	2	0
Nephroblastoma	2	0
Craniofacial germ cell tumor	1	0

**Table 3:** Microbiological and clinical characteristics of *Serratia* infections in oncohematological diseases (OHD) and hematopoietic stem cell transplantation (HSCT) groups.

	OHD Group (*n* = 30)	HSCT Group (*n* = 6)
Site of infection		
bloodstream	10	0
stool	8	1
urine	5	5
peritoneal fluid	2	0
insertion site	1	0
bronchoalveolar lavage	2	0
tracheal aspirate	1	0
catheter tip	1	0
Species		
*S. fonticola*	1	
*S. marcensens*	20	5
*S. liquefaciens*	7	0
*S. odonifera*	2	0
*S. ficaria*	0	1

**Table 4:** Causes of death in oncohematological diseases (OHD) group and hematopoietic stem cell transplantation (HSCT) group.

OHD Group		
Patient	Underlying diagnosis	Cause of death
1	AML	fulminant infection secondary to typhlitis with multiple organ dysfunction syndrome (not temporally associated with prior *Serratia* infection).
2	Brain tumor	progression
3	ALL	progression
4	MDS/AML	progression, multi organ failure in infection (not attributable to *Serratia* spp.)
5	AML	progression
HSCT group		
Patient 1	neuroblastoma	sepsis and veno-occlusive disease (culture negative)

## Data Availability

Requests to access the datasets should be directed to the corresponding author.

## Abbreviations

The following abbreviations are used in this manuscript:

ALL	acute lymphoblastic leukemia
AmpC	AmpC β-lactamase
BAL	bronchoalveolar lavage
CNS	central nervous system
ESBL	extended-spectrum β-lactamase
ESCMID	European Society of Clinical Microbiology and Infectious Diseases
HSCT	hematopoietic stem cell transplantation
IDSA	Infectious Diseases Society of America
NICU	neonatal intensive care unit
OHD	oncological hematological diseases
TMP/SMX	trimethoprim/sulfamethoxazole
UTI	urinary tract infection
